# Real-world characteristics and survival outcomes of patients with metastatic *ALK* fusion-positive solid tumors treated with standard-of-care therapies

**DOI:** 10.1093/oncolo/oyaf005

**Published:** 2025-05-08

**Authors:** Shirish M Gadgeel, Otto Fajardo, Fabrice Barlesi, Jeong Eun Kim, Razelle Kurzrock, David M Thomas, Ritika Jagtiani, Johannes Noe, Sven Schwemmers, Christos Nikolaidis

**Affiliations:** Department of Internal Medicine, Division of Hematology/Oncology, Henry Ford Cancer Institute/Henry Ford Health, Detroit, MI 48208, United States; Real World Data Science, Product Development Data Sciences, F. Hoffmann-La Roche Ltd, Basel 4070, Switzerland; Department of Medical Oncology, International Centre for Thoracic Cancers (CICT), Gustave Roussy, Villejuif 94800, France; Faculty of Medicine, Paris Saclay University, Kremlin-Bicêtre 94270, France; Department of Oncology, Asan Medical Centre, University of Ulsan College of Medicine, Seoul 05505, Republic of Korea; Precision Oncology Institution, Medical College of Wisconsin (MCW) Cancer Center, Milwaukee, WI 53226, United States; Medical Oncology, University of Nebraska, Omaha, NE 68182, United States; Worldwide Innovative Network (WIN) for Personalized Cancer Therapy, Chevilly-Larue 94550, France; Centre for Molecular Oncology, University of New South Wales, Sydney, New South Wales 2033, Australia; Product Development, Genentech, Inc., South San Francisco, CA 94080, United States; TM Oncology, F. Hoffmann-La Roche Ltd, Basel 4070, Switzerland; Global Product Development Medical Affairs (PDMA), F. Hoffmann-La Roche Ltd, Basel 4070, Switzerland; Product Development, F. Hoffmann-La Roche Ltd, Basel 4070, Switzerland

**Keywords:** anaplastic lymphoma kinase, ALK, prognostic, metastatic solid tumors

## Abstract

**Background:**

Anaplastic lymphoma kinase (*ALK*) fusions can be found in different solid tumors. This study aims to describe the clinical characteristics and investigate survival outcomes of patients with *ALK* fusion-positive solid tumors (excluding non-small cell lung cancer [NSCLC]) treated with standard-of-care therapies in a real-world setting.

**Patients and Methods:**

Data for patients with metastatic solid tumors (excluding NSCLC) who had ≥1 Foundation Medicine comprehensive genomic profiling (CGP) test between January 1, 2011 and September 30, 2023, were obtained from a nationwide (US-based) de-identified multi-tumor clinico-genomic database. Patients with *ALK* wild-type (*ALK*-WT) tumors were matched with patients with *ALK* fusion-positive tumors (4:1 ratio) using pre-specified baseline characteristics. Two models were used to analyze survival outcomes: Model 1 used the CGP report date as the index date; Model 2 used the date of metastatic diagnosis as the index date (including adjustment for immortal time bias).

**Results:**

Overall, 22 and 88 patients were included in the *ALK* fusion-positive and *ALK*-WT cohorts, respectively. Co-alterations were rare in the *ALK* fusion-positive cohort. Median overall survival was consistently lower in patients with *ALK* fusion-positive tumors compared with patients with *ALK*-WT tumors, across all analyses (hazard ratios between 1.8 and 2.0).

**Conclusion:**

Data from this study suggest that *ALK* fusions have a negative prognostic effect in metastatic solid tumors and highlight the need for further investigation of ALK inhibitors in the tumor-agnostic setting.

**Implications for Practice**

Non-small cell lung cancer (NSCLC) patients with anaplastic lymphoma kinase (*ALK*) fusions are routinely treated with ALK inhibitors. Preliminary data suggest that ALK inhibitors may also be efficacious across different tumor types. Nevertheless, *ALK* fusions are rare outside of NSCLC, which hinders the study of ALK inhibitors in randomized clinical trials. Our data show that *ALK* fusions have a negative prognostic effect in patients with metastatic solid tumors (other than NSCLC) treated with standard-of-care therapies in a real-world setting. These findings advocate for further research on the use of ALK inhibitors in patients with *ALK* fusion-positive solid tumors beyond NSCLC.

## Introduction

Anaplastic lymphoma kinase (*ALK*) fusions are critical oncogenic drivers of tumor growth and proliferation across a broad number of solid tumors but are considered to be rare. *ALK* alterations (comprising activating mutations, amplifications, and fusions/rearrangements) occur in ~3.3% of cancers.^1-3^ In contrast, *ALK* fusions/rearrangements are detected in ~0.2%-0.8% of cancers overall, with varying frequencies in different malignancies (3–7% of non-small cell lung cancer [NSCLC] vs 0.2% of non‐NSCLC tumors; >50% of inflammatory myofibroblastic tumors [IMTs] and anaplastic large cell lymphomas [ALCLs]).^1-4^

Crizotinib was the first ALK inhibitor to be approved for the treatment of *ALK* fusion-positive NSCLC, followed by ceritinib, alectinib, brigatinib, and lorlatinib.^3,5^ These ALK inhibitors have revolutionized the treatment of patients with *ALK* fusion-positive NSCLC, and are approved as front-line treatment options for these patients.^3,6-15^ Recent data have also demonstrated a clinical benefit for ALK inhibition in patients with *ALK* fusion-positive NSCLC in the adjuvant setting, resulting in the approval of alectinib in this setting.^10,16^

Outside of NSCLC, patients with *ALK* fusion-positive solid tumors are poorly served by current standard-of-care therapies, which have limited efficacy and/or significant off-target toxicity. Therefore, there is an unmet need for precision therapies that provide durable clinical benefits by selectively targeting *ALK* fusions and anticipated off-target resistance mechanisms. Evidence that *ALK* fusions are strong oncogenic drivers across tumor types suggests that ALK inhibitors may have tumor-agnostic activity; however, due to the rarity of *ALK* fusions, investigating the efficacy of ALK inhibitors in indications beyond NSCLC is challenging.^3^ Thus, the characteristics and natural history of patients with tumors harboring *ALK* fusions other than NSCLC have not been widely studied.

It is important to understand how survival outcomes differ between patients with *ALK* fusion-positive tumors versus patients with *ALK* wild-type (*ALK*-WT) tumors, under standard-of-care treatments, in order to determine whether *ALK* fusions may be clinically prognostic. This study was designed to broaden current knowledge of clinical characteristics and overall survival (OS) of patients with *ALK* fusion-positive solid tumors (excluding NSCLC) in a real-world setting, using data from clinical practice, and to characterize the prognostic value of the biomarker in the tumor-agnostic context.

## Patients and methods

### Study design and data source

This study was a retrospective analysis of clinical characteristics and survival outcomes of patients with *ALK* fusion-positive or *ALK*-WT solid tumors, using information from the nationwide (US-based) de-identified Flatiron Health-Foundation Medicine multi-tumor clinico-genomic database (CGDB; version September 2023). Retrospective, longitudinal clinical (patient-level structured and unstructured) data were derived from electronic health records (EHRs) and curated via technology-enabled abstraction. These were then linked to genomic data derived from the Foundation Medicine comprehensive genomic profiling (CGP) tests in the Flatiron Health-Foundation Medicine CGDB, using de-identified, deterministic matching.^17^ During the study period, de-identified data were obtained from ~280 Flatiron Health cancer clinics in the US (~800 care sites). As this study did not directly enroll patients, no ethics committee approval was required. The de-identified data were subject to obligations to prevent re-identification and protect patient confidentiality.

### Patient population

Patients were eligible for inclusion if they had ≥1 documented clinical visit in the Flatiron Health network between January 1, 2011 and September 30, 2023 and underwent CGP testing by Foundation Medicine before October 1, 2023 using one of Foundation Medicine’s solid tumor assays, FoundationOne or FoundationOneCDx.^18^ Only patients with a diagnosis of de novo Stage IV metastatic disease (Stage IV disease at diagnosis), who had not received an ALK inhibitor in any prior line of therapy, were considered for further analyses.

Patients with *ALK* fusion-positive NSCLC were excluded from this analysis since this is a well-studied population with several approved targeted therapies.^6-15^ Other exclusion criteria included treatment with an unlabeled study drug as part of a clinical trial, >1 CGP test, a visit gap of >90 days after initial diagnosis, multiple cancer diagnoses, no initial diagnosis date, a CGP report date before initial diagnosis, initial diagnosis within 3 months before data cut-off, and death before 2012 (year of Foundation Medicine CGP start).

### Determination of ALK status

Patients were considered *ALK* fusion-positive if their tumors had a 3’ *ALK* fusion with a protein-coding 5’ gene fusion partner, predicted to be in frame with an intact kinase domain. These fusions had a predicted known/likely functional status as defined by Foundation Medicine. Patients were deemed *ALK*-WT when no qualifying *ALK* alterations were observed by CGP.

### Covariate matching and statistical analyses

Patients with *ALK*-WT tumors were matched with patients with *ALK* fusion-positive tumors (4:1 ratio to minimize bias)^19^ using pre-selected baseline characteristics. Matching of patients within histological subtypes was conducted using the Mahalanobis distance method.^20^ Covariates used for matching included age, gender, race, tumor type, practice type (academic vs community), Eastern Cooperative Oncology Group performance status (ECOG PS) from 30 days before to 7 days after the index date, year of CGP, time from initial diagnosis to CGP report date, and number of lines of treatment prior to CGP report date. An absolute mean difference of <0.1 was used to indicate negligible differences between groups.^21^

Two models were used for the analysis: Model 1 used the CGP report date as the index date, whereas Model 2 used the date of metastatic diagnosis as the index date (including adjustment for immortal time bias using left truncation).^22,23^ Descriptive analyses were used to assess patient characteristics; assessments of frequencies were used for categorical variables, and means and standard deviations (SDs) for continuous variables. Overall survival was analyzed using the Kaplan-Meier method and Cox regression; medians and 95% confidence intervals (CIs) were calculated, along with hazard ratios (HRs) and associated 95% CIs. Sensitivity analyses were performed to assess the potential impact of tumor protein p53 (*TP53*) gene alterations (common in Stage IV cancers) on prognosis.

### Objectives

The primary objective of this study was to compare real-world OS in patients with *ALK*-WT versus *ALK* fusion-positive tumors, excluding NSCLC. OS was defined as the length of time in months from the index date until death from any cause or the censoring date (ie, last visit or encounter date). Secondary objectives included: description of patient characteristics and treatment patterns; characterization of *ALK* fusion partners and genetic alterations such as tumor mutational burden (TMB), microsatellite instability (MSI), and functional co-occurring alterations in actionable oncogenes (*RET*, *BRAF*, *ERBB2*, *EGFR*, *NTRK*, *ROS1*, *MET*, and *KRAS*), as well as other common alterations that manifest in the metastatic setting, such as *TP53* mutations.^24^

## Results

### Patient characteristics

**Figure 1.**
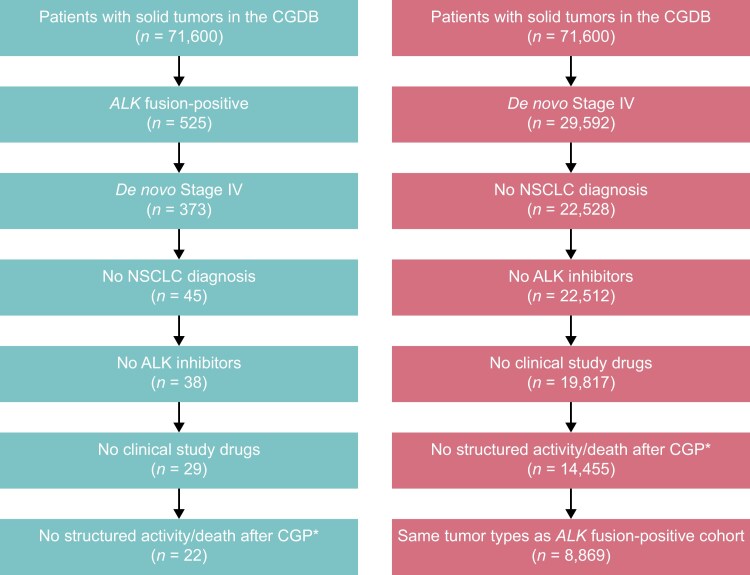
*ALK* fusion-positive and *ALK*-WT cohort attrition. *Also excluded patients with no initial metastatic disease diagnosis date or a diagnosis within 3 months before the data cut-off, patients who died before 2012, patients with multiple cancer diagnoses, patients with more than 1 CGP report, and patients with a CGP report date before the initial diagnosis. *ALK*, anaplastic lymphoma kinase; CGDB, clinico-genomic database; CGP, comprehensive genomic profiling; NSCLC, non-small cell lung cancer; WT, wild type.

Of the 525 patients with *ALK* fusion-positive solid tumors selected from the CGDB, 22 met the eligibility criteria and were included in the analysis (Figure 1). Of the 503 patients who did not meet the eligibility criteria, 152 were excluded for not having a de novo Stage IV diagnosis, and a further 328 were excluded because they had NSCLC. In the *ALK* fusion-positive cohort, the mean age was 62.6 years compared to 62.2 years in the matched *ALK-*WT cohort. Three patients (13.6%) had received ≥2 prior lines of therapy (Table 1).

**Table 1.** Baseline patient characteristics.

	*ALK* fusion-positive (*N* = 22)	*ALK*-WT (*N *= 8869)	
		Matched (*N* = 88)	Non-matched (*N* = 8,781)
Sex, *n* (%)			
Female	13 (59.1)	45 (51.1)	4163 (47.4)
Male	9 (40.9)	43 (48.9)	4618 (52.6)
Mean age, years (SD)^a^	62.6 (15.7)	62.2 (11.9)	62.7 (12.2)
Race, *n* (%)			
Asian	0	0	203 (2.3)
Black/African American	2 (9.1)	4 (4.5)	816 (9.3)
Hispanic/Latino	0	0	16 (0.2)
White	18 (81.8)	80 (90.9)	5623 (64.0)
Other/missing	2 (9.1)	4 (4.5)	2123 (24.2)
ECOG PS, *n* (%)			
0	5 (22.7)	26 (29.5)	2146 (24.4)
1	9 (40.9)	28 (31.8)	2816 (32.1)
2	0	0	822 (9.4)
≥3	1 (4.5)	4 (4.5)	237 (2.7)
Missing	7 (31.8)	30 (34.1)	2760 (31.4)
Number of prior lines of treatment, *n* (%)			
0	3 (13.6)	12 (13.6)	1247 (14.2)
1	7 (31.8)	32 (36.4)	3363 (38.3)
2	1 (4.5)	4 (4.5)	1120 (12.8)
≥3	2 (9.1)	8 (9.1)	1208 (13.7)
Missing	9 (40.9)	32 (36.4)	1843 (21.0)
Year of CGP report, *n* (%)			
<2019	4 (18.2)	16 (18.2)	3213 (36.6)
2019	2 (9.1)	8 (9.1)	1441 (16.4)
2020	6 (27.3)	25 (28.4)	1381 (15.7)
2021	6 (27.3)	25 (28.4)	1303 (14.8)
2022	3 (13.6)	10 (11.4)	1190 (13.6)
2023	1 (4.5)	4 (4.5)	253 (2.9)
Mean follow-up time from CGP report, months (IQR)	5.0 (16.8)	10.6 (16.9)	9.0 (16.1)
Mean time from initial diagnosis^b^ to CGP report date, months (IQR)	2.1 (5.6)	2.7 (9.8)	3.9(17.8)

^a^Patients with a birth year of 1938 or earlier may have an adjusted birth year in Flatiron datasets due to patient de-identification requirements.

^b^Of de novo metastatic disease.

*ALK*, anaplastic lymphoma kinase; CGP, comprehensive genomic profiling; ECOG PS, Eastern Cooperative Oncology Group performance status; IQR, interquartile range; SD, standard deviation; WT, wild type.

**Figure 2.**
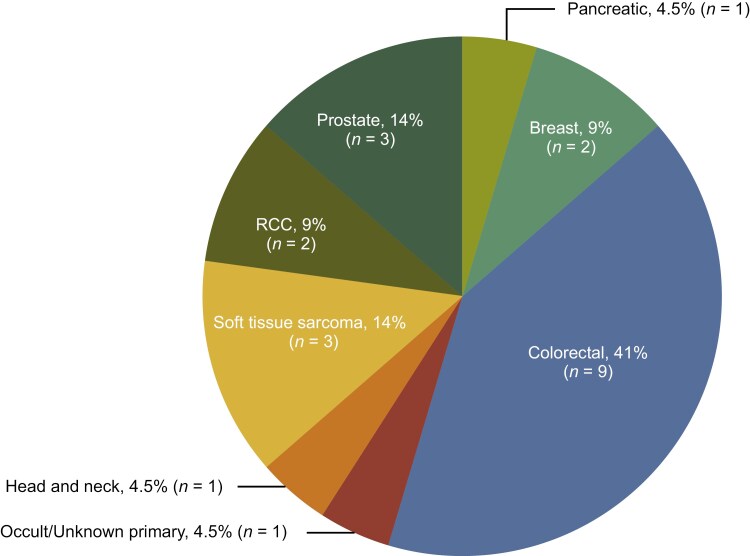
Tumor types in the *ALK* fusion-positive cohort (*N* = 22). Patients with *ALK* fusion-positive NSCLC were excluded. *ALK*, anaplastic lymphoma kinase; RCC, renal cell carcinoma.

The *ALK* fusion-positive cohort included 8 distinct tumor/histological subtypes, the most common of which were colorectal cancer (*n* = 9; 40.9%), sarcoma (*n* = 3; 13.6%), and prostate cancer (*n* = 3; 13.6%) (Figure 2). A total of 17 different *ALK* fusion partners were detected, of which the most common were *EML4* (*n* = 3; 13.6%), *STRN* (*n *= 3; 13.6%), and *SPTBN1* (*n* = 2; 9.1%); *ARHGAP15, ACTG2, CLIP4, CLTC, EPAS1, ERC1, FCHSD2, FN1, KIF5C, SLC8A1, SLMAP, SPINK5, TNS1,* and *ZNF143* were detected in 1 patient each.

A total of 8869 patients with *ALK*-WT solid tumors met the eligibility criteria. After covariate matching 4:1 with the *ALK* fusion-positive cohort (by patient characteristics, tumor type, ECOG PS, number of prior therapies, practice type, and timing of CGP), the matched *ALK*-WT cohort included 88 patients. The mean time from initial diagnosis to CGP report date was comparable between the *ALK*-positive and matched *ALK*-WT cohorts (2.1 [interquartile range (IQR) 5.6] and 2.7 [IQR 9.8] months, respectively; Table 1).

### Genomic alterations

Co-alterations were very rare in both the *ALK* fusion-positive and matched *ALK-*WT cohorts (Table 2), except for *KRAS* alterations in the matched *ALK-*WT cohort (*n* = 22; 25.0%). *TP53* alterations were equally common in both cohorts (50.0% in the *ALK* fusion-positive cohort and 44.3% in the matched *ALK*-WT cohort). TMB and MSI were also assessed, with few patients found to be TMB-high or MSI-high, and with a similar frequency in the 2 matched cohorts. However, ~30% of patients in the *ALK* fusion-positive cohort had unknown/missing TMB or MSI status (vs only 1%-2% of patients in the *ALK*-WT cohort).

**Table 2.** Co-occurring biomarkers^a^ and molecular characteristics.

	*ALK* fusion-positive (*N* = 22)	*ALK*-WT (*N* = 8869)	
		Matched (*N* = 88)	Non-matched (*N* = 8781)
TMB status, *n* (%)			
High (≥20 mut/Mb)	1 (4.5)	3 (3.4)	198 (2.3)
Medium (<20, ≥5.7 mut/Mb)	2 (9.1)	16 (18.2)	1286 (14.6)
Low (<5.7 mut/Mb)	13 (59.1)	68 (77.3)	7284 (83.0)
Missing	6 (27.3)	1 (1.1)	13 (0.1)
MSI-high, *n* (%)			
Yes	1 (4.5)	4 (4.5)	117 (1.3)
No	14 (63.6)	82 (93.2)	7700 (87.7)
Unknown/missing	7 (31.8)	2 (2.2)	964 (11.0)
Oncogenic alterations, *n* (%)			
*NTRK* rearrangement	0	0	16 (0.2)
*ROS1* rearrangement	0	0	12 (0.1)
*RET* rearrangement	0	0	11 (0.1)
*BRAF* alteration	0	5 (5.7)	424 (4.8)
*EGFR* alteration	1 (4.5)	1 (1.1)	45 (0.5)
*KRAS* alteration	1 (4.5)	22 (25.0)	3078 (35.1)
*ERBB2* amplification	1 (4.5)	1 (1.1)	353 (4.0)
*MET* alteration	0	0	17 (0.2)
*TP53* alteration	11 (50.0)	39 (44.3)	5359 (61.0)

^a^Only variants of “known” or “likely” functional status were included.

*ALK*, anaplastic lymphoma kinase; *BRAF*; v-raf murine sarcoma viral oncogene homolog B1; ERBB2, Erb-B2 receptor tyrosine kinase 2; *EGFR*, epidermal growth factor receptor; *KRAS*, kirsten rat sarcoma viral oncogene homologue; *MET*; mesenchymal epithelial transition factor; MSI, microsatellite instability; *NTRK*, neurotrophic tyrosine receptor kinase; *RET*, rearranged during transfection; *ROS1*, ROS proto-oncogene 1; TMB, tumor mutational burden; *TP53*, tumor protein p53; WT, wild type.

### Overall survival

**Figure 3.**
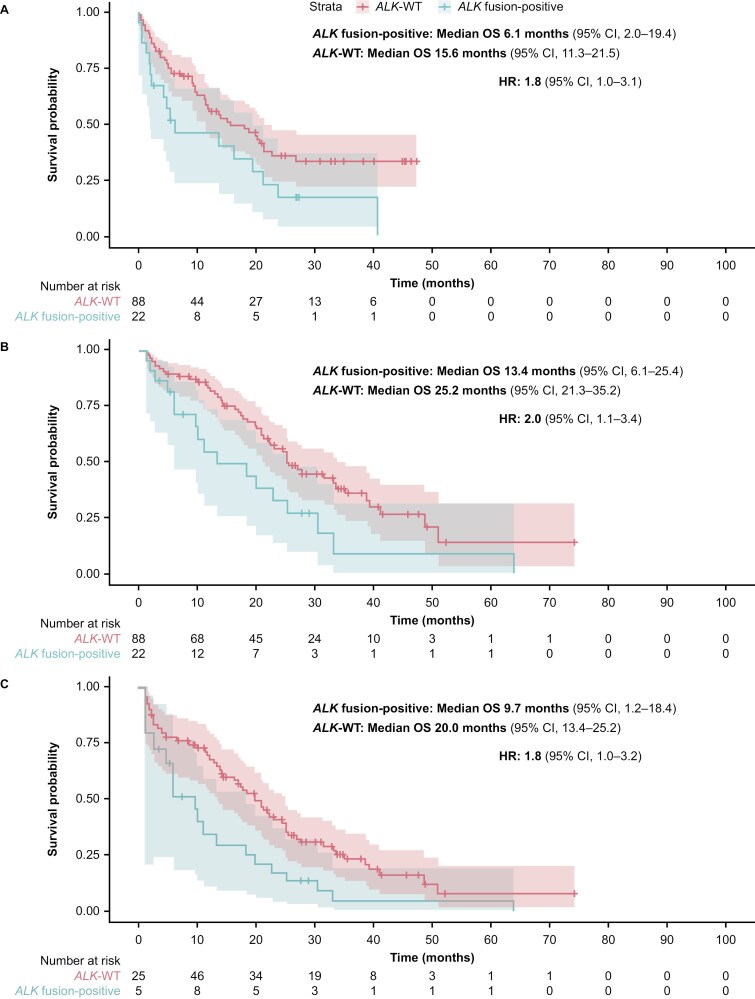
Kaplan-Meier plot estimates of OS comparing the *ALK* fusion-positive cohort (*N* = 22) with the matched *ALK*-WT cohort (*N* = 88) using (A) the CGP report date as the index (Model 1); (B) the initial diagnosis date as the index (Model 2); and (C) the initial diagnosis date as the index (Model 2*), and corrected for immortal time bias. *The number of patients is the same in all panels (ie, *N* = 22 for the *ALK* fusion-positive cohort and *N* = 88 for the *ALK*-WT cohort). Panel C shows the analysis using Model 2 and corrected for immortal time bias, which used left truncation to estimate survival. Only 5 patients with *ALK* fusion-positive tumors and 25 patients with *ALK*-WT tumors had both a metastatic diagnosis and a CGP date at time zero. For the remaining patients, who satisfied cohort entry criteria at later times, this immortal time was taken into account when calculating OS. *ALK*, anaplastic lymphoma kinase; CGP, comprehensive genomic profiling; CI, confidence interval; HR, hazard ratio; OS, overall survival; WT, wild type.

Overall survival was analyzed using 2 models. Model 1 used the CGP report date as the index whereas Model 2 used the date of initial diagnosis as the index, and included adjustment for immortal time bias. Results were consistent across the analyses (Table 3; Figure 3). When the CGP report date was used as the index (Model 1), median OS was 6.1 months (95% CI, 2.0-19.4) in the *ALK* fusion-positive cohort and 15.6 months (95% CI, 11.3-21.5) in the matched *ALK*-WT cohort; the HR was 1.8 (95% CI, 1.0-3.1). When the date of initial diagnosis was used as the index (Model 2), median OS was 13.4 months (95% CI, 6.1-25.4) in the *ALK* fusion-positive cohort and 25.2 months (95% CI, 21.3-35.2) in the matched *ALK*-WT cohort, with an HR of 2.0 (95% CI, 1.1-3.4). Lastly, when the date of initial diagnosis was used as the index (Model 2) and after adjusting for immortal time bias, median OS was 9.7 months (95% CI, 1.2-18.4) in the *ALK* fusion-positive cohort and 20.0 months (95% CI, 13.4-25.2) in the matched *ALK*-WT cohort, and the HR was 1.8 (95% CI, 1.0-3.2).

**Table 3.** Overall survival analysis by cohort.

		No. of deaths, *n* (%)	Median OS, months (95% CI)	HR, 95% CI
Model 1^a^	*ALK* fusion-positive cohort (*N* = 22)	17 (77.3%)	6.1 (2.0-19.4)	1.8 (1.0-3.1)
	Matched *ALK*-WT cohort (*N* = 88)	48 (54.5%)	15.6 (11.3-21.5)	
Model 2^b^	*ALK* fusion-positive cohort (*N* = 22)	17 (77.3%)	13.4 (6.1-25.4)	2.0 (1.1-3.4)
	Matched *ALK*-WT cohort (*N* = 88)	49 (55.7%)	25.2 (21.3-35.2)	
Model 2^b^ (corrected for immortal time bias)	*ALK* fusion-positive cohort (*N* = 22)	17 (77.3%)	9.7 (1.2-18.4)	1.8 (1.0-3.2)
	Matched *ALK*-WT cohort (*N* = 88)	49 (55.7%)	20.0 (13.4-25.2)	

^a^Model 1: using CGP report date as the index;

^b^Model 2: using date of initial diagnosis as the index.

*ALK*, anaplastic lymphoma kinase; CI, confidence interval; CGP, comprehensive genomic profiling; HR, hazard ratio; OS, overall survival; WT, wild type.

### Sensitivity analyses

**Figure 4.**
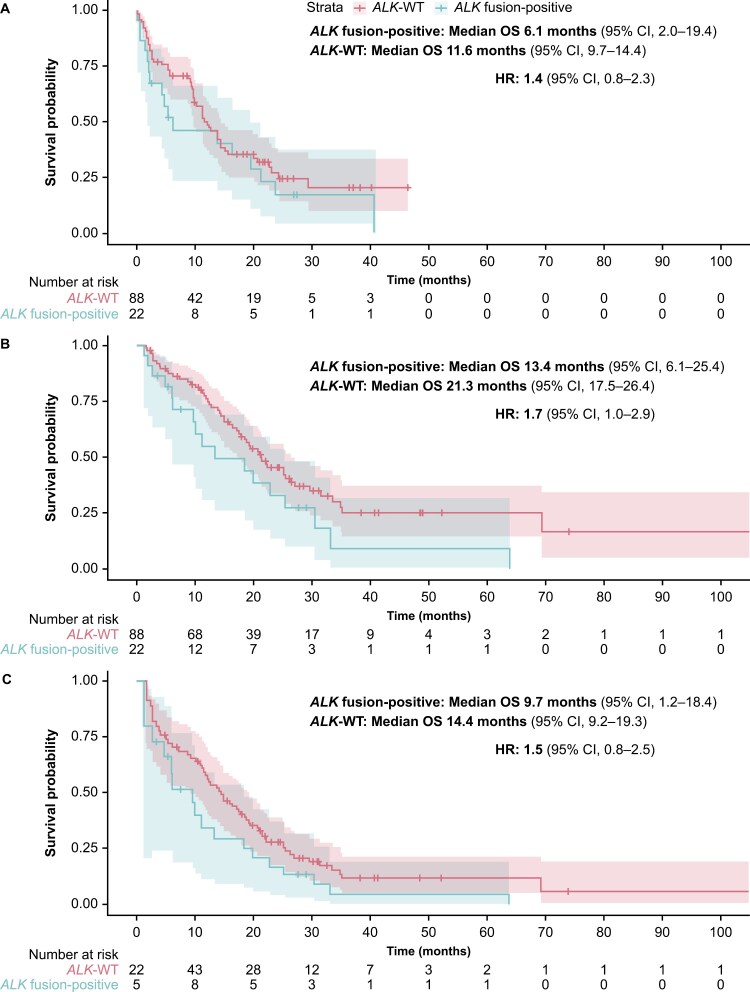
Sensitivity analyses: Kaplan-Meier estimates of OS analysis comparing the *ALK* fusion-positive cohort (*N* = 22) with the matched *ALK*-WT cohort (*N* = 88) and correcting for *TP53* alterations, using (A) the CGP report date as the index (Model 1); (B) the initial diagnosis date as the index (Model 2); and (C) the initial diagnosis date as the index (Model 2*), and corrected for immortal time bias. *The number of patients is the same in all panels (ie, *N* = 22 for the *ALK* fusion-positive cohort and *N* = 88 for the *ALK*-WT cohort). Panel C shows the analysis using Model 2 and corrected for immortal time bias, which used left truncation to estimate survival. Only 5 patients with *ALK* fusion-positive tumors and 22 patients with *ALK*-WT tumors had both a metastatic diagnosis and a CGP date at time 0. For the remaining patients, who satisfied cohort entry criteria at later times, this immortal time was taken into account when calculating OS. *ALK*, anaplastic lymphoma kinase; CGP, comprehensive genomic profiling; CI, confidence interval; HR, hazard ratio; OS, overall survival; *TP53*, tumor protein p53; WT, wild type.

Sensitivity analyses were performed to determine whether the presence of *TP53* co-alterations had an impact on the prognosis for these patients. After adjusting for the presence of *TP53* alterations, the median OS with Model 1 was 6.1 months (95% CI, 2.0-19.4) in the *ALK* fusion-positive cohort and 11.6 months (95% CI, 9.7-14.4) in the matched *ALK*-WT cohort; the HR was 1.4 (95% CI, 0.8-2.3) (Figure 4). With Model 2 after adjusting for the presence of *TP53* alterations, median OS was 13.4 months (95% CI, 6.1-25.4) in the *ALK* fusion-positive cohort and 21.3 months (95% CI, 17.5-26.4) in the matched *ALK*-WT cohort, with an HR of 1.7 (95% CI, 1.0-2.9). Lastly, with Model 2 after adjusting for immortal time bias and the presence of *TP53* alterations, median OS was 9.7 months (95% CI, 1.2-18.4) in the *ALK* fusion-positive cohort and 14.4 months (95% CI, 9.2-19.3) in the matched *ALK*-WT cohort, and the HR was 1.5 (95% CI, 0.8-2.5).

## Discussion

In this study, we investigated the clinical and demographic characteristics of patients with solid tumors harboring *ALK* fusions, and the prognostic value of *ALK* fusions. Co*-*alterations of common oncogenic drivers were rare in the *ALK* fusion-positive cohort (0%-4.5%), suggesting that *ALK* fusions were the main oncogenic drivers in this population. A shorter median OS was observed in patients with *ALK* fusion-positive versus *ALK*-WT tumors, with an HR of 1.8, suggesting that the risk of death for patients whose tumors harbor *ALK* fusions is almost double that of their wild-type counterparts (ie, *ALK* fusions have a negative prognostic effect). While patients with *ALK* fusion-positive NSCLC were excluded from this analysis, since this population is well characterized and there are already several approved targeted therapies in this setting, prior studies have also suggested that *ALK* rearrangements may be associated with worse disease outcomes in NSCLC.^25^ Genomic alterations in *TP53* were quite common in both the *ALK* fusion-positive and *ALK*-WT cohorts (50% and 44%, respectively) and data from sensitivity analyses suggest that *TP53* alterations, which typically manifest in the metastatic setting, may in general negatively affect the prognosis of these patients. Our data highlight the unmet need for patients with *ALK* fusion-positive solid tumors other than NSCLC, and warrant further investigation into the use of ALK inhibitors to improve their outcomes.

Several ALK TKIs are approved for patients with *ALK* fusion-positive NSCLC and crizotinib is also approved in patients with *ALK* fusion-positive relapsed or refractory, systemic ALCL and patients with *ALK* fusion-positive unresectable, recurrent or refractory IMTs.^6^ Investigating the efficacy of ALK inhibitors outside of NSCLC is challenging due to the rarity of *ALK* fusions,^3^ and this is supported by our findings; in our study, out of 22 528 patients with metastatic solid tumors (excluding NSCLC) in the CGDB, only 22 patients met the eligibility criteria for inclusion in the *ALK* fusion-positive cohort. These small numbers of patients means that randomized head-to-head trials would not be feasible, nor would it be possible to stratify patients (eg, by histology or other important patient characteristics, such as lines of prior treatments, tumor stage, etc.) in such studies. In addition, different tumor types might require different standards of care to be used as a comparator. Therefore, modified study designs (eg, single-arm basket or umbrella studies) are needed to generate meaningful data for tumor types with rare molecular alterations.^26,27^ Evidence suggests that ALK inhibitors may be efficacious across different indications^3,28^ and there is a need for further investigations of the clinical value of ALK TKIs in a tumor-agnostic way. The signal-seeking MyPathway trial (NCT02091141)^28^ completed in 2023 and additional studies to investigate ALK TKIs in adult and pediatric patients in a tumor-agnostic setting are currently ongoing or completed (eg, TAPISTRY platform study [NCT04589845]^29^; iMATRIX study [NCT04774718]^30^). In our study, patients with *ALK* fusion-positive tumors treated with standard-of-care therapies had poor survival outcomes, suggesting that this genomic alteration is not a favorable prognostic factor. Therefore, any potential benefit seen with ALK inhibitors in these tumor-agnostic studies would most likely be due to the treatment itself, and not confounded by the presence of the *ALK* alteration.

Similar analyses using real-world data have recently been published for patients with *NTRK* fusion-positive or *RET* fusion-positive solid tumors, which concluded that both *NTRK* and *RET* fusions are also negative prognostic factors.^22,31,32^ Real-world data are used to evaluate patient outcomes based on the presence of certain oncogenic drivers and could therefore be considered as complementary source of evidence (eg, in patients with rare molecular alterations).

Our study has several strengths. We used real-world data from a large database and drawn from EHRs, thus reflecting the nuances of routine clinical practice, and spanning more than a decade of observations. This longitudinal follow-up provides insights into the durability of treatment responses, late effects of therapy, and real-world survival outcomes, beyond the limited follow-up periods typical of clinical trials. Moreover, it allows the exploration of heterogeneity in treatment effects across different patient populations, thus enhancing the generalizability of findings to real-world clinical practice. The use of 2 models for analyzing OS also supports the robustness of our findings. Lastly, there is an established track record of studies investigating the prognostic effect of precision oncology biomarkers, including *NTRK* fusions^31,32^ and *RET* fusions^22^ in solid tumors, using a similar methodology.

This study also has some limitations. This was a retrospective analysis with a small number of patients with *ALK* fusion-positive tumors and, although this is reflective of the rare nature of *ALK* fusions,^4^ it still limits our ability to draw definitive conclusions. Additionally, since the study used a heterogeneous population, it is unclear if tumor types with relatively long survival/large censoring may have impacted the data, despite the fact that only de novo metastatic tumors (with equally poor prognosis) were included in the analyses, and that underlying patient and clinical characteristics were successfully matched. In addition, clinical characteristics were not always available, with data regarding the number of prior lines of therapy missing for more than a third of patients. Furthermore, since CGP is not yet routinely used across all tumor types, it is not known how representative the patient population is of the wider population of patients with *ALK* fusion-positive solid tumors. There may also be potential bias if genetic testing was preferentially performed on some patients for reasons that were not captured in this analysis.

In conclusion, our data suggest a negative prognostic value for *ALK* fusions in metastatic solid tumors, highlighting an unmet medical need for precision therapies that provide durable clinical benefit by selectively targeting *ALK* fusions. Additional analyses with larger patient populations must be conducted to assess the validity of these findings in the future.

## Data Availability

For up-to-date details on Roche’s Global Policy on the Sharing of Clinical Information and how to request access to related clinical study documents, see here: https://go.roche.com/data_sharing. The data that support the findings of this study were originated by and are the property of Flatiron Health, Inc. and Foundation Medicine, Inc., which has restrictions prohibiting the authors from making the data set publicly available. Requests for data sharing by license or by permission for the specific purpose of replicating results in this manuscript can be submitted to PublicationsDataaccess@flatiron.com and cgdb-fmi@flatiron.com.

## References

[1] AACR Project GENIE Consortium. AACR Project GENIE: powering precision medicine through an international consortium. Cancer Discov. 2017;7:818-831. 28572459 10.1158/2159-8290.CD-17-0151 PMC5611790

[2] Della Corte CM , Viscardi G, Di Liello R, et al Role and targeting of anaplastic lymphoma kinase in cancer. Mol Cancer. 2018;17:30. https://doi.org/10.1186/s12943-018-0776-2 29455642 PMC5817803

[3] Shreenivas A , Janku F, Gouda MA, et al ALK fusions in the pan-cancer setting: another tumor-agnostic target? NPJ Precis Oncol. 2023;7:101. https://doi.org/10.1038/s41698-023-00449-x 37773318 PMC10542332

[4] Ross JS , Ali SM, Fasan O, et al ALK fusions in a wide variety of tumor types respond to anti-ALK targeted therapy. Oncologist. 2017;22:1444-1450. https://doi.org/10.1634/theoncologist.2016-0488 29079636 PMC5728036

[5] Schrank Z , Chhabra G, Lin L, et al Current molecular-targeted therapies in NSCLC and their mechanism of resistance. Cancers (Basel) 2018;10:224. https://doi.org/10.3390/cancers10070224 29973561 PMC6071023

[6] Administration UFD. Crizotinib FDA prescribing information. 2023. Accessed April 22, 2024. https://www.accessdata.fda.gov/drugsatfda_docs/label/2023/202570s036lbl.pdf

[7] European Medicines Agency (EMA). Crizotinib summary of product characteristics. Accessed April 22, 2024. https://www.ema.europa.eu/en/documents/product-information/xalkori-epar-product-information_en.pdf

[8] Administration UFD. Ceritinib FDA prescribing information. 2021. Accessed April 22, 2024. https://www.accessdata.fda.gov/drugsatfda_docs/label/2021/205755s019lbl.pdf

[9] European Medicines Agency (EMA). Ceritinib summary of product characteristics. Accessed April 22, 2024. https://www.ema.europa.eu/en/documents/product-information/zykadia-epar-product-information_en.pdf

[10] Administration UFD. Alectinib FDA prescribing information. 2024. Accessed April 22, 2024. https://www.accessdata.fda.gov/drugsatfda_docs/label/2024/208434s015lbl.pdf

[11] European Medicines Agency (EMA). Alectinib summary of product characteristics. Accessed April 22, 2024. https://www.ema.europa.eu/en/documents/product-information/alecensa-epar-product-information_en.pdf

[12] Administration UFD. Brigatinib FDA prescribing information. 2022. Accessed April 22, 2024. https://www.accessdata.fda.gov/drugsatfda_docs/label/2022/208772s013lbl.pdf

[13] European Medicines Agency (EMA). Brigatinib summary of product characteristics. Accessed April 22, 2024. https://www.ema.europa.eu/en/documents/product-information/alunbrig-epar-product-information_en.pdf

[14] Administration UFD. Lorlatinib FDA prescribing information. 2021. Accessed April 22, 2024. https://www.accessdata.fda.gov/drugsatfda_docs/label/2021/210868s004lbl.pdf

[15] European Medicines Agency (EMA). Lorlatinib summary of product characteristics. Accessed April 22, 2024. https://www.ema.europa.eu/en/documents/product-information/lorviqua-epar-product-information_en.pdf

[16] Wu YL , Dziadziuszko R, Ahn JS, et al; ALINA Investigators. Alectinib in resected ALK-positive non-small-cell lung cancer. N Engl J Med. 2024;390:1265-1276. https://doi.org/10.1056/NEJMoa2310532 38598794

[17] Singal G , Miller PG, Agarwala V, et al Association of patient characteristics and tumor genomics with clinical outcomes among patients with non-small cell lung cancer using a clinicogenomic database. JAMA. 2019;321:1391-1399. https://doi.org/10.1001/jama.2019.3241 30964529 PMC6459115

[18] Frampton GM , Fichtenholtz A, Otto GA, et al Development and validation of a clinical cancer genomic profiling test based on massively parallel DNA sequencing. Nat Biotechnol. 2013;31:1023-1031. https://doi.org/10.1038/nbt.2696 24142049 PMC5710001

[19] Linden A , Samuels SJ. Using balance statistics to determine the optimal number of controls in matching studies. J Eval Clin Pract. 2013;19:968-975. https://doi.org/10.1111/jep.12072 23910956

[20] Rubin DB. Bias reduction using Mahalanobis-metric matching. Biometrics. 1980;36:293-298. https://doi.org/10.2307/2529981

[21] Harder VS , Stuart EA, Anthony JC. Propensity score techniques and the assessment of measured covariate balance to test causal associations in psychological research. Psychol Methods. 2010;15:234-249. https://doi.org/10.1037/a0019623 20822250 PMC2936698

[22] Hackshaw A , Fajardo O, Dafni U, et al Characteristics and survival outcomes of patients with metastatic RET fusion-positive solid tumors receiving non-RET inhibitor standards of care in a real-world setting. JCO Precision Oncol. 2024;8:e2300334. https://doi.org/10.1200/PO.23.00334 PMC10830092 38271655

[23] Mackenzie T. Survival curve estimation with dependent left truncated data using Cox’s model. Int J Biostat. 2012;8:/j/ijb.2012.8.issue-/j/ijb.2012.81/1557. https://doi.org/10.1515/1557-4679.1312 23104845

[24] Pandey R , Johnson N, Cooke L, et al *TP53* mutations as a driver of metastasis signaling in advanced cancer patients. Cancers 2021;13:597. https://doi.org/10.3390/cancers13040597 33546249 PMC7913278

[25] Chaft JE , Dagogo-Jack I, Santini FC, et al Clinical outcomes of patients with resected, early-stage ALK-positive lung cancer. Lung Cancer (Amsterdam, Netherlands) 2018;122:67-71. https://doi.org/10.1016/j.lungcan.2018.05.020 30032847 PMC6062851

[26] Le Tourneau C , André F, Helland A, et al Modified study designs to expand treatment options in personalised oncology: a multistakeholder view. Eur J Cancer (Oxford, England : 1990) 2023;194:113278. https://doi.org/10.1016/j.ejca.2023.113278 37820553

[27] Di Liello R , Piccirillo MC, Arenare L, et al Master protocols for precision medicine in oncology: overcoming methodology of randomized clinical trials. Life (Basel, Switzerland) 2021;11:1253. https://doi.org/10.3390/life11111253 34833129 PMC8618758

[28] Swanton C , Friedman CF, Sweeney CJ, et al Activity and safety of alectinib for ALK-altered solid tumors from MyPathway. Cancer Res. 2022;82:CT032-CT032. https://doi.org/10.1158/1538-7445.am2022-ct032

[29] Drilon AE , Liu H, Wu F, et al Tumor-agnostic precision immuno-oncology and somatic targeting rationale for you (TAPISTRY): a novel platform umbrella trial. J Clin Oncol. 2021;39:TPS3154-TPS3154. https://doi.org/10.1200/jco.2021.39.15_suppl.tps3154

[30] Doz F , Casanova M, Koh K-N, et al Abstract CT039: alectinib in children and adolescents with solid or CNS tumors harboring ALK-fusions: data from the iMATRIX Alectinib Phase I/II open-label, multi-center study. Cancer Res. 2024;84:CT039-CT039. https://doi.org/10.1158/1538-7445.am2024-ct039

[31] Bazhenova L , Lokker A, Snider J, et al TRK fusion cancer: patient characteristics and survival analysis in the real-world setting. Target Oncol. 2021;16:389-399. https://doi.org/10.1007/s11523-021-00815-4 33893941 PMC8105201

[32] Hibar DP , Demetri GD, Peters S, et al Real-world survival outcomes in patients with locally advanced or metastatic NTRK fusion-positive solid tumors receiving standard-of-care therapies other than targeted TRK inhibitors. PLoS One. 2022;17:e0270571. https://doi.org/10.1371/journal.pone.0270571 35939431 PMC9359555

